# DeepDicomSort: An Automatic Sorting Algorithm for Brain Magnetic Resonance Imaging Data

**DOI:** 10.1007/s12021-020-09475-7

**Published:** 2020-07-05

**Authors:** Sebastian R. van der Voort, Marion Smits, Stefan Klein, for the Alzheimer’s Disease Neuroimaging Initiative

**Affiliations:** 1 Biomedical Imaging Group Rotterdam, Departments of Radiology and Nuclear Medicine and Medical Informatics, Erasmus MC - University Medical Centre Rotterdam, Rotterdam, The Netherlands; 2 Department of Radiology and Nuclear Medicine, Erasmus MC - University Medical Centre Rotterdam, Rotterdam, The Netherlands

**Keywords:** DICOM, Brain imaging, Machine learning, Magnetic resonance imaging, BIDS, Data curation

## Abstract

With the increasing size of datasets used in medical imaging research, the need for automated data curation is arising. One important data curation task is the structured organization of a dataset for preserving integrity and ensuring reusability. Therefore, we investigated whether this data organization step can be automated. To this end, we designed a convolutional neural network (CNN) that automatically recognizes eight different brain magnetic resonance imaging (MRI) scan types based on visual appearance. Thus, our method is unaffected by inconsistent or missing scan metadata. It can recognize pre-contrast T1-weighted (T1w),post-contrast T1-weighted (T1wC), T2-weighted (T2w), proton density-weighted (PDw) and derived maps (e.g. apparent diffusion coefficient and cerebral blood flow). In a first experiment,we used scans of subjects with brain tumors: 11065 scans of 719 subjects for training, and 2369 scans of 192 subjects for testing. The CNN achieved an overall accuracy of 98.7%. In a second experiment, we trained the CNN on all 13434 scans from the first experiment and tested it on 7227 scans of 1318 Alzheimer’s subjects. Here, the CNN achieved an overall accuracy of 98.5%. In conclusion, our method can accurately predict scan type, and can quickly and automatically sort a brain MRI dataset virtually without the need for manual verification. In this way, our method can assist with properly organizing a dataset, which maximizes the shareability and integrity of the data.

## Introduction

With the rising popularity of machine learning, deep learning, and automatic pipelines in the medical imaging field, the demand for large datasets is increasing. To satisfy this hunger for data, the amount of imaging data collected at healthcare institutes keeps growing, as is the amount of data that is shared in public repositories (Greenspan et al. 2016; Lundervold and Lundervold 2019). However, this increase in available data also means that proper data curation, the management of data throughout its life cycle, is needed to keep the data manageable and workable (Prevedello et al. 2019; van Ooijen 2019). One essential data curation step is organizing a dataset such that it can easily be used and reused. Properly organizing the dataset maximizes the shareability and preserves the full integrity of the dataset, ensuring repeatability of an experiment and reuse of the dataset in other experiments.

Unfortunately, the organization of medical imaging data is not standardized, and the format in which a dataset is provided often differs between sources (Lambin et al. 2017; van Ooijen 2019). Efforts such as the brain imaging data structure (BIDS) (Gorgolewski et al. 2016) propose a standardized data structure, to which some public data repositories adhere (e.g. OpenNeuro Gorgolewski et al. 2017, ABIDE Martino et al. 2017 and OASIS LaMontagne et al. 2018). However, other repositories do not conform to this standard (e.g. The Cancer Imaging Archive (TCIA) Clark et al. 2013, Alzheimer’s Disease Neuroimaging Initiative (ADNI), and PPMI Marek et al. 2018). Furthermore, similar to some prospectively collected research data, retrospectively collected data from clinical practice usually do not follow a standardized format either (van Ooijen 2019). Thus, the challenge of structuring a dataset, either into a BIDS compliant dataset or a different format, remains.

When using a medical imaging dataset in a research project, one needs to select the scan types that are relevant to the research question (Montagnon et al. 2020; Lambin et al. 2017). Thus, it is essential to identify the scan type of each scan when sorting a medical imaging dataset. Different data sources do not use consistent naming conventions in the metadata of a scan (e.g. the series description), which complicates the automatic identification of the scan type (van Ooijen 2019; Wang et al. 2011). Moreover, in some cases, this metadata is not consistently stored (e.g. contrast administration Hirsch et al. 2015) and might even be partially or entirely missing, as can be the case for anonymized data (Moore et al. 2015). As a result, the sorting is frequently done manually, by looking at each scan and labeling it according to the perceived scan type. This manual labeling can be a very time-consuming task, which hampers scientific progress; thus, it is highly desirable to automate this step of the data curation pipeline. Similar arguments concerning the complexity of medical imaging data and the importance of data structuring also motivated the creation of the BIDS standard (Gorgolewski et al. 2016).

Previous research has focused on modality recognition (Dimitrovski et al. 2015; Yu et al. 2015; Arias et al. 2016), as well as on distinguishing different modalities of (MRI) scans (Srinivas and Mohan 2014; Remedios et al. 2018). Only one of these studies (Remedios et al. 2018) considered the prediction of the scan type of (MRI) scans, who predicted 4 sacn types, namely precontrast T1-weighted (T1w), post-contrast T1-weighted (T1wC), fluid-attenuated inversion recovery (FLAIR) and T2-weighted (T2w) scans. However, with the increasing popularity of multi-parametric (MRI) in machine learning algorithms and automatic pipelines (Li et al. 2017; Akkus et al. 2017; Nie et al. 2016; Pereira et al. 2015), the need to recognize more sacn types is arising.

In this research, we propose a method, called DeepDicomSort, that recognizes eight different sacn types of brain (MRI) scans, and facilitates sorting into a structured format. DeepDicomSort is a pipeline consisting of a pre-processing step to prepare scans as inputs for a convolutional neural network (CNN), a scan type recognition step using a CNN, and a post-processing step to sort the identified sacn types into a structured format. Our method identifies T1w, T1wC, T2w, proton density-weighted (PDw), T2-weighted fluid-attenuated inversion recovery (T2w-FLAIR), diffusionweighted imaging (DWI) (including trace/isotropic images), perfusion-weighted dynamic susceptibility contrast (PWI-DSC) scans, and diffusion-weighted and perfusion-weighted derived maps (including, for example, apparent diffusion coefficient (ADC), fractional anisotropy, and relative cerebral blood flow). Once the sacn types have been identified, DeepDicomSort can organize the dataset into a structured, user-defined layout or turn the dataset into a BIDS compliant dataset. We made all our source code, including code for the pre-processing and post-processing, and pre-trained models publicly available, to facilitate reuse by the community.^1^

## Materials & Methods

### Terminology

Since the exact meaning of specific terms can differ depending on one’s background, we have provided an overview of the terminology as it is used in this paper in Table 1. We have tried to adhere to the terminology used by BIDS as much as possible, and have provided the equivalent BIDS terminology in Table 1 as well. We differ from the BIDS terminology regarding two terms: scan and scan type. Scan type is referred to as modality in BIDS, but to avoid confusion with the more common use of modality to indicate different types of equipment (e.g. (MRI) and computed tomography (CT)), we instead use scan type. Scan is used instead of “data acquisition” or “run” as used in BIDS, to be more in line with common terminology and to avoid confusion with other types of data acquisition. We define a structured dataset as a dataset where all the data for the different subjects and scans is provided in the same way. For example, a folder structure with a folder for each subject, session and scan with a consistent naming format for the different folders and sacn types. A standardized dataset is a dataset where the data has been structured according to a specific, public standard, for example BIDS.

**Table 1:** Overview of terminology used in this paper, the corresponding BIDS terminology and meaning of each term

Term	BIDS term	Meaning
Modality	Modality	Type of technique used to acquire a scan (e.g. (MRI), CT)
Subject	Subject	A person participating in a study
Site	Site	Institute at which a scan of the subject has been acquired
Session	Session	A single visit of a subject to a site in which one or more scans have been acquired
Scan	Data acquisition/run	A single 3D image that has been acquired of a subject in a session
Slice	N/A	A single 2D cross-section that has been extracted from a scan
Scan type	Modality	Specific visual appearance category of a scan (e.g. T1w, T2w))
Sample	N/A	A single input for the CNN
Class	N/A	An output category of the CNN
DICOM	DICOM	A data format used to store medical imaging data. In addition to the imaging data, DICOM files can also store metadata about the scanner equipment, the specific imaging protocol and clinical information.
NIfTI	NIfTI	A data format used to store (neuro) medical imaging data.

### Data

An extensive collection of data from multiple different sources was used to construct our method and evaluate its performance. We used (MRI) scans of subjects with brain tumors, as well as scans of subjects without brain tumors.

To ensure sufficient heterogeneity in our dataset, we included scans from multiple different sources, and we only excluded scans if their scan type did not fall into one of the eight categories that we aimed at predicting with our method. Thus, no scans were excluded based on other criteria such as low image quality, the occurrence of imaging artifacts, scanner settings, or disease state of the subject.

#### Brain Tumor Dataset

Our method was initially developed and subsequently tested on brain (MRI) scans of subjects with brain tumors. Scans of subjects with brain tumors were used because the brain tumor imaging protocols used to acquire these scans usually span a wide array of sacn types, including pre-contrast and post-contrast scans. The brain tumor dataset consisted of a train set and an independent test set, which in total included data from 11 different sources. The subjects were distributed among the brain tumor train set and brain tumor test set before starting any experiments, and the data was divided such that the distribution of the sacn types was similar in the train set and the test set. We chose to put all subjects that originated from the same dataset in either the train set or test set to test the generalizability of our algorithm. Thus, all scans of a subject were either all in the brain tumor train set or all in the brain tumor test set, and no data leak could take place, precluding an overly optimistic estimation of the performance of our method. In this way, a good performance of our method on the test set could not be the result of the algorithm having learned features that are specific to a particular site or scanner.

The brain tumor train set contained 11065 scans of 1347 different sessions from 719 subjects. These scans were included from the Brain-Tumor-Progression (Schmainda and Prah 2018), Ivy Glioblastoma Atlas Project (Ivy GAP) (Shah et al. 2016), LGG-1p19qDeletion (Erickson et al. 2016; Akkus et al. 2017), TCGA-GBM (Scarpace et al. 2016) and TCGA-LGG (Pedano et al. 2016) collections from TCIA (Clark et al. 2013). Two datasets from The Norwegian National Advisory Unit for Ultrasound and Image Guided Therapy (USIGT) (Fyllingen et al. 2016; Xiao et al. 2017) were also included in the brain tumor train set. In total, the data originated from 17 different sites, and the scans were acquired on at least 29 different scanner models from 4 different vendors (GE, Hitachi, Philips, and Siemens).

The brain tumor test set contained 2369 scans of 302 different sessions from 192 subjects. These scans were included from the brain images of tumors for evaluation (BITE) dataset (Mercier et al. 2012) as well as the Clinical Proteomic Tumor Analysis Consortium Glioblastoma Multiforme (CPTAC-GBM) (National Cancer Institute Clinical Proteomic Tumor Analysis Consortium (CPTAC) 2018), Repository of Molecular Brain Neoplasia Data (REMBRANDT) (Scarpace et al. 2015), and Reference Image Database to Evaluate Therapy Response: Neuro MRI (RIDER Neuro MRI) (Barboriak 2015) collections from the TCIA. In total, the data originated from 8 different sites, and the scans were acquired on at least 15 different scanner models from 4 different vendors (GE, Philips, Siemens, and Toshiba).

For some scans, the scanner type was not available in the DICOM tags (DICOM tag (0008, 1090)); thus, the data variation in the number of scanners could be even larger.

All subjects included in the brain tumor dataset had a (pre-operative or post-operative) brain tumor. The scans in the datasets were manually sorted, and T1w, T1wC, T2w, PDw, T2w-FLAIR, DWI, PWI-DSC, and derived images were identified. The different types of derived images were combined into a single category, as the derivation of these images is often inconsistent among scanners and vendors, and thus these images need to be rederived from the raw data (e.g. the original DWI or PWI-DSC scan).

**Fig. 1:**
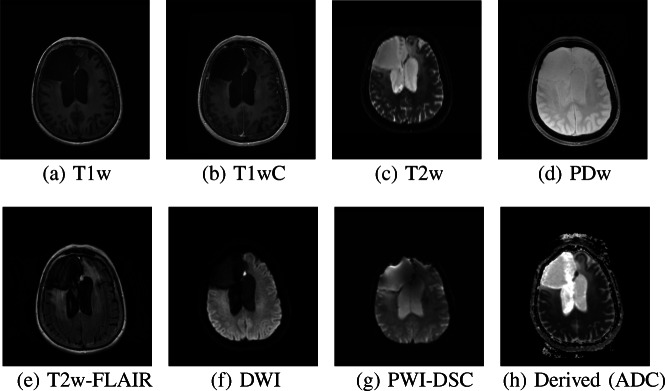
Examples of the different sacn types for a single subject from the brain tumor test set

The details of the brain tumor train set and brain tumor test set are presented in Table 2. An example of the eight sacn types for a single subject from the brain tumor test set can be seen in Fig. 1.

**Table 2:** Overview of data in the brain tumor dataset

	Brain tumor train set					Brain tumor test set				
Scan type	Ax	Cor	Sag	3D	Total	Ax	Cor	Sag	3D	Total
T1w	580	14	872	454	1920	206	2	202	26	436
T1wC	964	526	298	1040	2828	208	133	97	172	610
T2w	1151	411	23	31	1616	232	46	16	1	295
PDw	413	40	0	0	453	145	36	0	0	181
T2w-FLAIR	991	39	4	50	1084	221	3	0	32	256
DWI	1359	0	0	0	1359	347	0	0	0	347
PWI-DSC	669	0	0	0	669	87	0	0	0	87
Derived	1136	0	0	0	1136	157	0	0	0	157
Total	7263	1030	1197	1575	11065	1603	220	315	231	2369

The number of scans for each scan type and the different spatial orientations (axial, coronal, sagittal and 3D) are specified

#### ADNI Dataset

In order to evaluate the results of the algorithm on non-tumor brain imaging, we used the ADNI dataset (adni.loni.usc.edu). The ADNI was launched in 2003 as a public-private partnership, led by Principal Investigator Michael W. Weiner, MD. The primary goal of ADNI has been to test whether serial magnetic resonance imaging, positron emission tomography, other biological markers, and clinical and neuropsychological assessment can be combined to measure the progression of mild cognitive impairment and early Alzheimer’s disease. For up-to-date information, see adni-info.org.

We used the baseline and screening data of 1318 subjects, resulting in 7227 scans. These scans originated from 67 different sites and were acquired on 23 different scanner models from 3 different vendors (GE, Philips, and Siemens). Details of the ADNI dataset are presented in Table 3. Since no contrast is administered to subjects in the ADNI study, there are no T1wC or PWI-DSC scans in this dataset. The ADNI dataset does include arterial spin labeling perfusion-weighted imaging (PWIASL), however since our algorithm was not designed to recognize these scans, they were excluded. The derived maps from these PWI-ASL scans were included since the derived category encompasses all diffusion and perfusion derived imaging. These PWI-ASL derived maps explain the 47 3D scans in Table 3.

**Table 3:** Overview of data in the ADNI dataset

	ADNI dataset				
Scan type	Ax	Cor	Sag	3D	Total
T1w	0	0	276	2380	2656
T1wC	0	0	0	0	0
T2w	1725	488	5	0	2218
PDw	1069	0	0	0	1069
T2w-FLAIR	1	0	3	488	492
DWI	558	0	2	0	560
PWI-DSC	0	0	0	0	0
Derived	183	0	2	47	232
Total	3536	488	288	2915	7227

The number of scans for each scan type and the different spatial orientations (axial, coronal, sagittal and 3D) are specified

### DeepDicomSort

The pipeline of our proposed method, DeepDicomSort, consisted of three phases:

1. Pre-processing: prepare the scans as an input for the CNN
2. Scan type prediction: obtain the predicted scan type using the CNN
3. Post-processing: use the predictions to sort the dataset

**Fig. 2:**
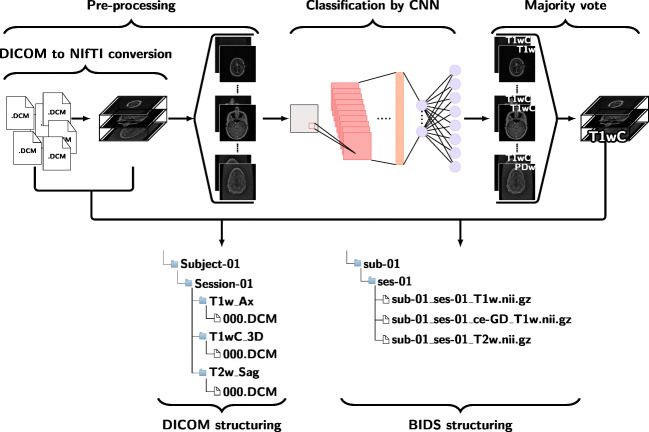
Overview of the DeepDicomSort pipeline. Scans are first converted from DICOM to NIfTI format and pre-processed. During the pre-processing the scan is split into 25 individual slices, that are then classified as one of eight sacn types by the CNN. The predictions of the individual slices are combined in a majority vote and the predicted scan type of each scan is used to structure the dataset. DeepDicomSort can structure either the original DICOM files, or the NIfTI files. In the last case the dataset turns into BIDS compliant dataset

By passing a dataset through this pipeline, it can be turned into a BIDS compliant dataset, or it can be structured according to a user-defined layout. If one chooses to create a BIDS compliant dataset, the scans are stored as NIfTI files; if a user-defined structure is used, the scans are stored as DICOM files. An overview of the DeepDicomSort pipeline is presented in Fig. 2.

#### Pre-Processing

As a first pre-processing step, all DICOM files were converted to NIfTI format using dcm2niix (Li et al. 2016), as this simplifies the further processing of the scans. This step was skipped for the USIGT and BITE datasets, as these were already provided in NIfTI format (no DICOM files were available).

In the next step, the number of dimensions of each scan was automatically determined. Although most scans were 3-dimensional, some scans happened to be 4-dimensional. This was the case for some DWI scans, which consisted of multiple b-values and potentially b-vectors, and for some PWI-DSC scans, which contained multiple time points. If a scan was 4-dimensional, the first (3D) element of the sequence was extracted and was subsequently used instead of the full 4-dimensional scan. This extraction was done to make sure that the CNN would also recognize sacn types that generally contain repeats in situations where this was not the case. For example, this could be the case when the different b-values of a DWI scan were stored as multiple, separate (3D) scans instead of a single (4D) scan. Since the information that a scan is 4-dimensional can aid the algorithm in recognizing the scan type, a “4D” label was attached to each scan. This 4D label was set to 1 if the scan was 4-dimensional, and to 0 if it was not.

All scans were then reoriented to match the orientation of a common template using FSL’s reorient2std (Jenkinson et al. 2012). After this step, the scans were resampled to 256 × 256 × 25 voxels, using cubic b-spline interpolation, while maintaining the original field of view. All of these resampled (3D) scans were split into (2D) slices, resulting in 25 individual slices of 256 × 256 voxels. The slice extraction was then followed by an intensity scaling of each slice. The intensity was scaled such that the minimum intensity was 0, and the maximum intensity was 1 to compensate for intensity differences between slices. These pre-processed slices were than used as input samples for the CNN. No data augmentation was used, as the large number of scans and different data sources that were used to train the algorithm already ensured sufficient natural variation in the samples, obviating the need for additional augmentation.

After applying these pre-processing steps, the brain tumor train set consisted of 276625 samples, the brain tumor test set consisted of 59225 samples, and the ADNI dataset consisted of 180675 samples.

#### Network

**Fig. 3:**
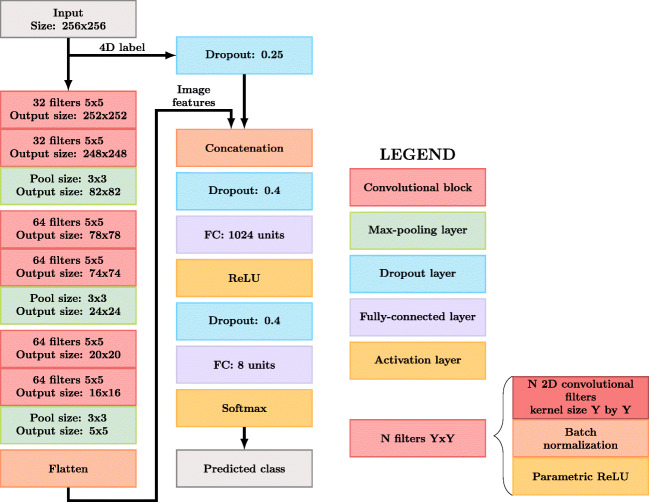
The architecture of the CNN. The convolutional blocks consisted of N 2D convolutional filters followed by batch normalization and a parametric rectified linear unit. The output size of the convolutional blocks and pooling layers is specified

A CNN was used to classify the samples into one of eight different classes: T1w, T1wC, T2w, PDw, T2w-FLAIR, DWI, PWI-DSC, or derived. The architecture of the CNN is shown in Fig. 3. This architecture was inspired by the VGG network (Simonyan and Zisserman 2015).

**Fig. 4:**
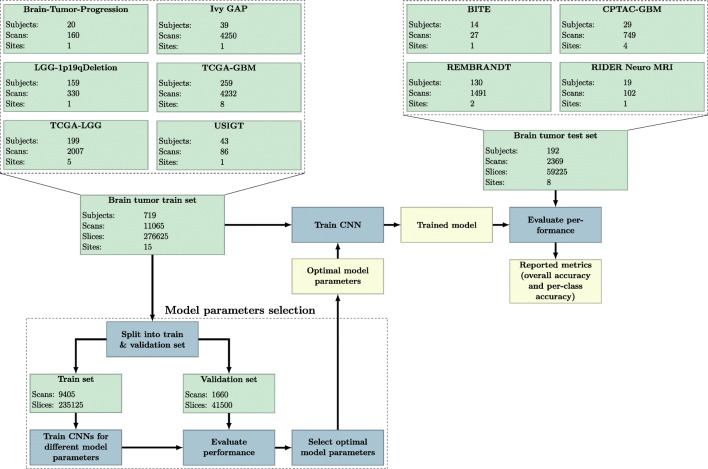
Overview of Experiment I. In this experiment, the brain tumor train set was used to obtain the optimal model parameters and to train the algorithm. The trained model was then evaluated on the brain tumor test set

The network was implemented using TensorFlow 1.12.3 (Abadi et al. 2016). The cross-entropy between the predicted and ground truth labels was used as a loss function. Weights were initialized using Glorot Uniform initialization (Glorot and Bengio 2010). We used Adam as an optimizer (Kingma and Ba 2015), which started with a learning rate of 0.001, β_1_ = 0.9, and β_2_ = 0.999, as these were proposed as reasonable default values (Kingma and Ba 2015). The learning rate was automatically adjusted based on the training loss; if the training loss did not decrease during 3 epochs, the learning rate was decreased by a factor 10, with a minimum learning rate of 1 ⋅ 10^− 7^. The network could train for a maximum of 100 epochs, and the network automatically stopped training when the loss did not decrease during 6 epochs. We used a batch size of 32. We arrived at this CNN design and these settings by testing multiple different options and selecting the best performing one. Details about the optimization of the settings are presented in Section “Experiments”, Fig. 4, and Appendix A.

During the training of the network, all slices were inputted to the CNN as individual samples, and no information about the (possible) relation between different slices was provided. After training the network, the scan type of a scan was predicted by passing all 25 slices of the scan through the CNN and then combining these individual slice predictions using a majority vote.

#### Post-Processing

Once the scan type of each scan is predicted, these predictions can then be used in (optional) post-processing steps to automatically structure the dataset. We provide two options for the structured format:

- Sort the original DICOM files; this can be done in a user-defined folder structure.
- Sort the NIfTI files; in this case the BIDS format is used.

During the post-processing, the spatial orientation of the scan (axial, coronal, sagittal, or 3D) is also determined based on the direction cosines (DICOM tag (0020, 0037)), which can be used to define the structured layout when choosing to sort the DICOM files.

### HeuDiConv

HeuDiConv^2^ is a heuristic-centric DICOM converter, which uses information from the DICOM tags, along with a user-defined heuristic file to organize an unstructured DICOM dataset into a structured layout. HeuDiConv is currently one of the most widespread, publicly available methods that can structure an unsorted DICOM dataset. Therefore, we used HeuDiConv as a benchmark so we could compare our method, which is based on the visual appearance of a scan, with a method that is based on the metadata of a scan.

Before HeuDiConv can be used to sort a dataset, one first needs to define the heuristic file, which is essentially a translation table between the metadata of a scan and its scan type. This heuristic file is based on scan metadata that is extracted from the DICOM tags. Available metadata includes image type, study description, series description, repetition time, echo time, size of the scan along 4 dimensions, protocol name, and sequence name. HeuDiConv also determines whether a scan is motion-corrected or is a derived image, based on specific keywords being present in the image type DICOM tag. These characteristics can also be used in the heuristic file. Although more scan metadata can be used to define the heuristic, such as subject gender and referring physician, we considered this metadata irrelevant for our purpose of scan type prediction. In addition, this kind of metadata was often missing due to anonymization.

### Experiments

#### Evaluation of DeepDicomSort

We performed two experiments in which we constructed and evaluated our method, to show the generalizability among different datasets:

- Experiment I: Algorithm trained on brain tumor train set and tested on brain tumor test set
- Experiment II: Algorithm trained on brain tumor dataset (brain tumor train set and brain tumor test set), and tested on ADNI dataset

In Experiment I we developed the algorithm and tried different CNN architectures, pre-processing settings, and optimizer settings, collectively referred to as the model parameters, using a train/validation split of the brain tumor train set. We then selected the best performing model parameters and trained a CNN using the whole brain tumor train set. Once the model was trained, its performance was evaluated on the brain tumor test set. In Experiment I, the brain tumor test set was only used to evaluate the results and was left untouched during the development and training of the algorithm. Figure 4 shows an overview of the model parameter selection, training and testing steps, and the data used in Experiment I. More details about the selection of the optimal model parameters and the results of other model parameters can be found in Appendix A.

**Fig. 5:**
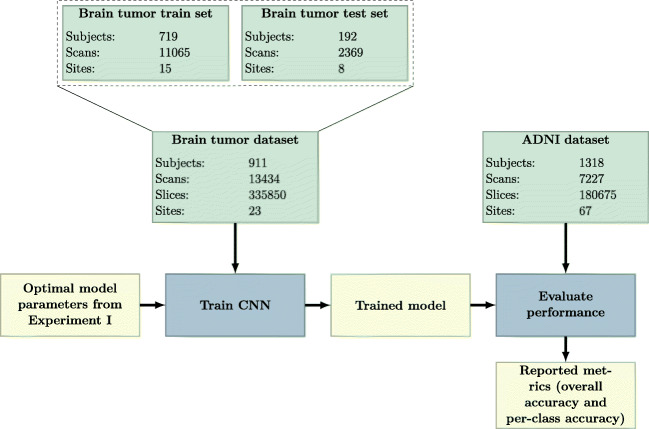
Overview of Experiment II. In this experiment the brain tumor dataset was used to train the algorithm, and the trained model was then evaluated on the ADNI dataset

In Experiment II we used the ADNI dataset as a test set to see if our method also generalizes to scans in which no brain tumor was present. In this experiment, we trained the CNN using the whole brain tumor dataset (a combination of all the data in the brain tumor train set and brain tumor test set) and then evaluated the performance of the model on the ADNI dataset. No model parameter selection was done in this experiment, instead the optimal model parameters that were obtained from Experiment I were used. Thus, apart from training the CNN on a larger dataset, the methods used in Experiment I and Experiment II were the same. Figure 5 shows an overview of the training and testing steps and the data used in Experiment II. In this experiment, no T1wC and PWI-DSC scans were present in the test set, however in a real-world setting one may not know a priori whether these sacn types were present or absent. Thus, we still allowed the model to predict the scan type as one of these classes to mirror this realistic setting.

To evaluate the performance of our algorithm, we calculated the overall accuracy and the per-class accuracy of the classification. The overall accuracy was defined as the number of correctly predicted scans divided by the total number of scans. The per-class accuracy was defined as the number of correctly predicted scans of a specific scan type divided by the total number of scans of that scan type. We also computed the confusion matrices, which show the relationship between the ground truth and predicted class.

To visualize which parts of the slice contributed most to the prediction of the CNN, we generated saliency maps (Simonyan et al. 2014). Saliency maps were generated by calculating the gradient of a specific class with respect to each input pixel, thus giving a measure of the contribution of each pixel. To obtain sharper maps, we used guided backpropagation (Springenberg et al. 2015) and applied a rectified linear activation to the obtained maps. Saliency maps were generated for all slices of the scans of the example subject shown in Fig. 1, based on the trained model from Experiment I. Additional saliency maps were generated for 20 samples of each scan type that were randomly selected from the test sets of Experiment I and Experiment II. The saliency maps for the samples from Experiment I were generated using the CNN trained in Experiment I, and for the samples from Experiment II the CNN trained in Experiment II was used. By generating saliency maps for multiple samples, we could show the behavior of our algorithm for different scan appearances. Some of these samples contained tumors, contained imaging artifacts or had a low image quality. Thus, these saliency maps also showed the robustness of our algorithm to unusual scan appearance. To gain some insight into the behavior of each convolutional layer we determined the feature maps of each convolutional layer. We calculated the feature maps for the T1w slice shown in Fig. 1 by passing it through the network and determining the output of each filter after each convolutional layer.

#### Comparison with HeuDiConv

We compared the performance of HeuDiConv and DeepDicomSort using the data from Experiment I, since the data in Experiment II did not include all sacn types. When using HeuDiConv, only the scans which were available in DICOM format could be processed. This meant that the scans from the USIGT dataset were removed from the brain tumor train set, and the scans from the BITE dataset were removed from the brain tumor test set, as these were not available in DICOM format. Thus, 86 scans (43 T1wC and 43 T2w-FLAIR) were removed from the brain tumor train set and 27 scans (all T1wC) were removed from the brain tumor test set, reducing the train set to 10979 scans and the test set to 2342 scans.

**Fig. 6:**
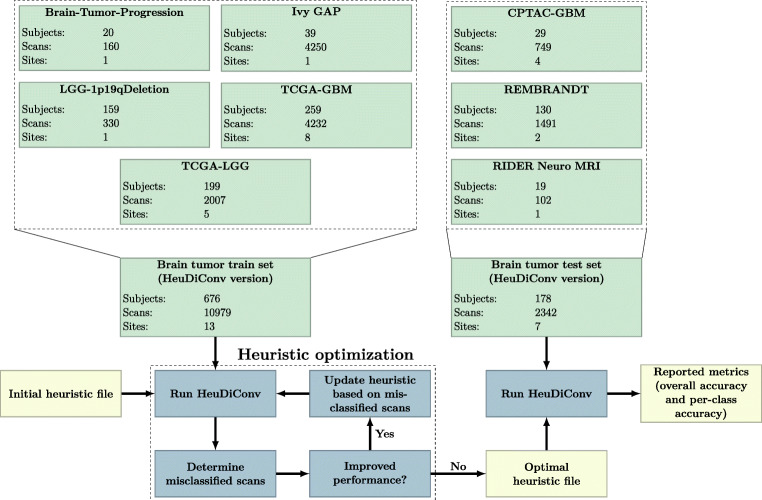
Overview of the HeuDiConv experiment. In this experiment the scans from the brain tumor train set that were available in DICOM format were used to construct the heuristic file. HeuDiConv used this heuristic file to predict the scan type of the scans from the brain tumor test set which were available in DICOM format

To construct our heuristic, we first extracted all the relevant DICOM tags from the scans in the brain tumor train set, see Table 4. Table 4 also shows the number of unique occurrences for text-based tags and the distribution of the numerical tags in the brain tumor train set. An iterative approach was followed to construct the heuristic, where rules were added or adjusted until the performance of HeuDiConv on the brain tumor train set could no longer be increased, see Fig. 6. Our initial heuristic was a simple one, based solely on certain text being present in the series description. For example, if the text “T1” was present in the series description, it was considered a T1w scan.

**Table 4:** DICOM tag numbers and descriptions of the DICOM tags extracted for the HeuDiConv heuristic

Tag description	Tag number	
Image type	0008,0008	72 unique instances
Study description	0008,1030	435 unique instances
Series description	0008,103E	1215 unique instances
Repetition time	0018,0080	Mean ± std: 3912 ± 4078
Echo time	0018,0081	Mean ± std: 52.11 ± 48.9
Number of rows in image	0028,0010	Range: 128 - 1152
Number of columns in image	0028,0011	Range: 128 - 1152

For text-based tags the number of unique instances is shown and for numerical-based tags the distribution is shown, based on the scans in the brain tumor train set

To compare the performance of HeuDiConv with the performance of DeepDicomSort the overall accuracy and per-class accuracy of the scan type predictions obtained from HeuDiConv were calculated.

## Results

### Experiment I - Evaluation on Brain Tumor Dataset

The results from Experiment I (evaluation on the brain tumor test set, containing scans of subjects with brain tumors) are reported in Table 5. The network was trained for 96 epochs. In this experiment our method achieved an overall accuracy of 98.7%.

**Table 5:** Overall accuracy and per-class accuracy achieved by DeepDicomSort in Experiment I and Experiment II

	Experiment I	Experiment II
*Overall*	0.987	0.985
*T1w*	0.993	1.000
*T1wC*	0.997	N/A
*T2w*	0.990	0.965
*PDw*	1.000	0.998
*T2w-FLAIR*	0.930	0.951
*DWI*	0.991	0.995
*PWI-DSC*	1.000	N/A
*Derived*	0.994	0.983

The highest per-class accuracy was achieved for the PDw and PWI-DSC scans (100.0% for both), whereas the T2w-FLAIR scans had the lowest accuracy (93.0%). The confusion matrices show that most of the incorrectly predicted T2w-FLAIR scans were classified as T1w scans (see Appendix B). Appendix C shows the performance of our method on a per-slice basis before the majority vote has taken place to determine the scan class, which shows that the per-slice accuracy is lower than the per-scan accuracy. This is not surprising since there are slices in a scan from which it is almost impossible to determine the scan type even for a human (for example, the most superior and inferior slices).

### Experiment II - Evaluation on ADNI Dataset

The results from Experiment II (evaluation on the ADNI dataset, containing scans of subjects without brain tumors) are reported in Table 5. Just like in Experiment I the network was trained for 96 epochs. In this experiment our method achieved an overall accuracy of 98.5%. It took approximately 22 hours to train the network of this experiment using an Nvidia Titan V GPU with 12 GB memory.

The highest per-class accuracy was achieved for the T1w scans (100.0%), whereas the T2w scans had the lowest accuracy (95.1%). Most of the incorrectly predicted T2w scans were predicted as T1wC or PDw scans. Furthermore, although no T1wC and PWI-DSC scans were present in the test set used in this experiment, our method incorrectly classified 40 scans as T1wC (mainly T2w scans) and 3 scans as PWI-DSC scans (all DWI scans). The full confusion matrix can be found in Appendix B.

### Focus of the Network

**Fig. 7:**
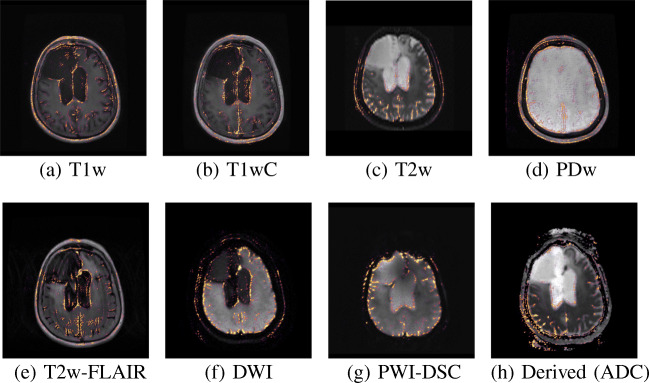
Saliency maps of the sacn types, generated by the CNN evaluated on the same slices as in Fig. 1. This CNN was the model obtained in Experiment I

Figure 7 shows the saliency maps for the different sacn types, for the same slices as in Fig. 1. For most sacn types, the CNN seemed to focus on the ventricles, the cerebral spinal fluid (CSF) around the skull, the nose, and the eyes. For the PDw slice, the CNN did not have a specific focus on the ventricles and did not seem to have a particular focus inside the brain. The DWI and derived slices also showed some focus outside of the skull, probably because of the artifacts outside of the skull that these sacn types often feature (as can be seen in Fig. 7h). We have created saliency maps for all 25 slices of the scans shown in Fig. 1, which are shown in Appendix E. For most other slices the focus of the CNN was the same as for the slices from Fig. 7. Furthermore, the presence of a tumor did not disturb the prediction as also evidenced by the high accuracy achieved in Experiment I. Only on the most superior and inferior slices did the CNN struggle, probably due to the fact that the brain was barely visible on those slices.

Additional saliency maps for randomly selected samples from the test sets of Experiment I and Experiment II are shown in Appendix F. These examples show that our method is robust to heterogeneity in the visual appearance of the scans, as well as to the presence of tumors, the presence of imaging artifacts, and poor image quality. This is demonstrated by the fact that the CNN focused on the same brain structures for almost all of the slices and correctly predicted the scan type even for slices with poor imaging quality or artifacts. The feature maps of all convolutional layers are shown in Appendix G. For the shallow convolutional layers, some filters seemed to detect the skull without looking at the brain tissue, whereas other layers seemed to focus more on specific brain structures such as the CSF. Interpreting the deeper convolutional layers gets harder as the feature maps of those layers have a lower resolution.

### HeuDiConv Predictive Performance

The top-level rules of the derived heuristic for HeuDiConv were mainly based on the series description, with additional lower-level rules based on the echo time, image type, and the derived status of the scan. The overall accuracy obtained within the brain tumor train set after several iterations of improving the heuristic was 91.0%. The overall accuracy in the brain tumor test set was 72.0%. The results for each class can be found in Table 6, along with a comparison to the accuracy of the CNN evaluated on the brain tumor test set. For the evaluation of the CNN’s performance, we included the same scans as present in the test set for HeuDiConv (i.e. those which were available in DICOM format). Although a slightly different dataset was used for this test set, the results of the CNN in Tables 8 and 6 appear to be the same. This can be explained by the fact that only T1wC scans were removed from the test set, thus for all other classes the accuracy remained the same. Furthermore, due to the large number of scans the difference is only visible at more decimals, e.g. the overall accuracy in Table 8 was 98.73% whereas in Table 6 it was 98.72%. These results show that DeepDicomSort outperformed HeuDiConv both in terms of the overall accuracy and the per-class accuracy for all classes. Appendix D compares the time required to sort the datasets using either DeepDicomSort, HeuDiConv, or by hand, which shows that DeepDicomSort is more than twice as fast as the other two methods.

**Table 6:** Accuracy of HeuDiConv on the brain tumor test set

	HeuDiConv	DeepDicomSort
*Overall*	0.720	0.987
*T1w*	0.963	0.993
*T1wC*	0.447	0.997
*T2w*	0.930	0.990
*PDw*	0.077	1.000
*T2w-FLAIR*	0.684	0.930
*DWI*	0.887	0.991
*PWI-DSC*	0.600	1.000
*Derived*	0.948	0.994

Results of DeepDicomSort on this test set are also given, where the scans which were not available in the DICOM format were excluded from the test set

## Discussion

Our results show that it is possible to use a CNN to automatically identify the scan type of brain (MRI) scans and use this to sort a large, heterogeneous dataset. Because of the high accuracy of our method, it can be used virtually without manual verification. The CNN performed well both for scans with and without the presence of a tumor. The performance of our method generalizes well across scans from different sites, scanners, subjects, and scan protocols. Our method was also able to correctly predict the scan type of scans that had poor imaging quality or contained imaging artifacts, as can be seen in Appendix F.1. The CNN focused mainly on the ventricles, areas close to the skull, and the CSF at the edges of the brain. There was also some focus on the gray matter and white matter, although these structures seemed less relevant for the decision making of the CNN. It makes sense that the CNN focuses on the CSF, both in the ventricles and at the edges of the brain, because their visual appearance is very characteristic of the scan type. Although the CNN also focused on the eyes and nose, we do not expect this to disrupt the prediction when these structures are absent (e.g. in defaced scans). There were a lot of slices in which the eyes and nose were not present, such as the most inferiorly and superiorly located slices, for which the CNN predicted the scan type correctly.

Data sorting is just one step of the data curation pipeline, and in recent years more research on the automation of other data curation tasks has been carried out. Some examples include automatic scan quality checking (Esteban et al. 2017), motion artifact correction (Tamada et al. 2020), and missing scan type imputation from the present sacn types (Lee et al. 2019). However, to automate other data curation steps the dataset first needs to follow a structured format, making our tool a crucial first step in the overall pipeline. The increasing data complexity, both in volume and in the number of different types of data, not only shows a need for a proper data curation pipeline, but also shows the need for a standardized data structure for scans and their associated metadata (van Erp et al. 2011; Gorgolewski et al. 2016; Lambin et al. 2017). The widespread adoption of a common, standardized data structure would be favorable over the use of our tool or similar tools. Unfortunately, both in research and in clinic practice, it is currently not commonplace to provide datasets in a standardized format, thus making our tool a valuable addition to the data curation pipeline. Even if a standardized data structure were to be widely adopted, our tool would remain valuable as a quality assessment tool.

**Fig. 8:**
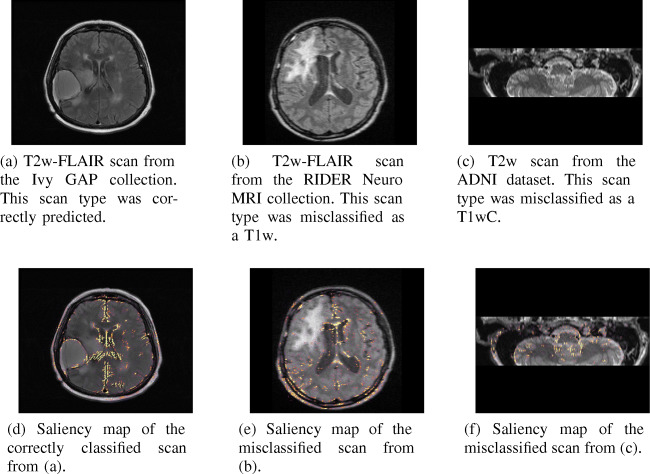
Examples of scans our method misclassified (**b** and **c**) and a correctly classified scan (**a**) as comparison, along with their saliency maps. The T2w-FLAIR scan in (**b**) is probably misclassified as its appearance is very different from T2w-FLAIR scans that were in the train dataset. The T2w scan in (**c**) is probably misclassified because it has a very limited field of view

Although the accuracy of our method is high overall, our method predicted the incorrect scan type in some cases. For example, in Experiment I the CNN mainly misclassified T2w-FLAIR scans. Almost all of these misclassified T2w-FLAIR scans originated from the RIDER Neuro MRI dataset. Comparing a T2w-FLAIR scan from the RIDER dataset with a T2w-FLAIR scan from the train set used in Experiment I shows a big difference in visual appearance, see Fig. 8a and b. These figures show that the white matter and gray matter appear very different on the two scans, even though they have the same scan type, which probably confused the network. In Experiment II the per-class accuracy was the lowest for the T2w scans. Almost all of the misclassified T2w scans were hippocampus scans, an example of which can be seen in Fig. 8c. The misclassification of these scans can be explained by their limited field of view. Since the CNN did not see any such scans in the training set, as all scans in the training set covered the full brain, it is not surprising that our method failed in these cases. The saliency maps in Fig. 8 show that the CNN had difficulty focusing on the relevant parts of the slice. For example, for the T2w-FLAIR slices in Figs. 7e and 8e it can be seen that the CNN focused mainly on the ventricles, whereas in Fig. 8e there was more focus on the edge of the brain, similar to T1w slice in Fig. 7a. Although we did not achieve a perfect prediction accuracy, it is unlikely that any scan sorting method ever will, due to the large heterogeneity in scan appearance and scan metadata. While not perfect, our method does have a very high performance overall and the comparison with manual sorting shows that it considerably reduces the time required to sort a dataset.

The CNN was trained and evaluated by using the ground truth labels, which were obtained by manually going through the dataset and annotating each scan according to the perceived scan type. It is possible that the scan type was incorrectly annotated for some of the scans. To limit this possibility we took a second look at scans where there was a mismatch between the prediction from DeepDicomSort and the ground truth label, both for train datasets and test datasets. We corrected the ground truth label for scans that were incorrectly annotated and these corrected labels were used for the experiments presented in this paper. The labels of around 0.1% of the scans in the dataset were corrected in this way. Although it is possible that there were still some incorrectly annotated scans, based on these findings we expect this fraction to be very small.

We chose a CNN as the basis of our method because we wanted to minimize the number of pre-processing steps. Using more traditional machine learning approaches, such as a support vector machine or random forest, would require the extraction of relevant features from each scan. This would complicate our method as we would first have to hand-craft these features and add a pre-processing step in which we extract these features from the scan. Furthermore, the extraction of these features would likely require a brain mask to prevent the features from being influenced too much by the background. The creation of this brain mask would add a pre-processing step, and could be a potential source of error. Instead, by using a CNN, no features had to be defined as the CNN automatically learns the relevant features. The CNN also does not require a brain mask, as it has learned to ignore the background and focus on the brain itself, as shown by the saliency maps.

We opted for a 2D CNN instead of a 3D CNN, because this allowed us to extract a larger region of the scan to be used as an input for the CNN. By using a 2D CNN, this region could encompass a full slice of the brain enabling the CNN to learn features that capture the relative differences in appearance of the various tissue types (white matter, gray matter, CSF, bone, skin, etc.), which are characteristic of the scan type. Furthermore, because a 2D CNN typically requires less memory than a 3D CNN (Prasoon et al. 2013), it requires less computational power (making our method accessible to a broader audience), and also requires less time to train and evaluate (Li et al. 2014).

Our method achieved a better overall accuracy and per-class accuracy than HeuDiConv. The results obtained using HeuDiConv show the difficulty of creating a method based on DICOM tags that generalizes well to other datasets. Even within one dataset, it can be difficult to create a heuristic that correctly maps the scan metadata to the scan type; for example Table 4, shows that 1215 different series descriptions are used just for the eight sacn types considered in this research. HeuDiConv has particular difficulty in identifying scans that have similar metadata but have different sacn types. For example, this is reflected in the results for the T1w and T1wC scans. These scans usually have similar scan settings and series descriptions, making it hard to determine whether a scan is obtained pre- or post-contrast administration. The same difficulty plays a role for T2w and PDw scans, which are often acquired at the same time in a combined imaging sequence and thus have the same series description. In our timing results (Appendix D), it was faster to sort the dataset by hand than to use HeuDiConv. This was caused by HeuDiConv often misclassifying T2w-FLAIR and T1wC scans as a different scan type, and thus a lot of manual time was needed to correct these mistakes.

A method that, similar to ours, classifies the scan type based on the visual appearance of the scan was proposed by Remedios et al. (2018) called Φ-net. Their method can identify T1w, T1wC, T2w, and pre-contrast and post-contrast FLAIR scans. Remedios et al. do this using a cascaded CNN approach where a first CNN is used to classify a scan as T1-weighted, T2-weighted, or FLAIR. Two other CNNs are then used to classify a scan as pre-contrast or post-contrast, one CNN for the T1-weighted scans and one CNN for the FLAIR scans. Φ-net achieved an overall accuracy of 97.6%, which is lower than our overall accuracy of 98.7% (Experiment I) and 98.5% (Experiment II). Since Remedios et al. did not make their trained model publicly available, it was not possible to directly compare performances on the same dataset. Remedios et al. tested their method on 1281 scans, which came from 4 different sites and 5 different scanner models. Their dataset was thus considerably smaller and less heterogeneous than our test data set. Furthermore, our method can identify more sacn types and does so using only a single CNN instead of three.

A limitation of our method is that it can only classify a scan as one of the eight sacn types for which it was trained. Thus, when it is presented with an unknown scan type (e.g. PWI-ASL or dynamic contrast-enhanced perfusion-weighted imaging), our method will (wrongly) predict it as one of the other classes. In future work, this limitation could be addressed in two ways. The first option would be to adapt the network to either recognize more sacn types or to replace one of the existing classes by a different one. This can be done using a transfer learning approach by fine-tuning the weights obtained in this research on additional data (Tajbakhsh et al. 2016). Since we did not have enough data for other sacn types, we limited the CNN to the eight classes for which we did have enough data. A second option would be to extend our method to allow out-of-distribution detection (DeVries and Taylor 2018). In this methodology, the network could not only predict the scan type of a scan but could also indicate if a scan belongs to an unknown scan type. This requires a significant change to the model architecture, which we considered outside the scope of this research for now.

Another limitation is the use of reorient2std from FSL, which means that (this part of) the code cannot be used in a commercial setting. Commercially allowed alternatives exist, such as the ’reorient_image’ function from ANTs (http://stnava.github.io/ANTs/), however these have not been tested as part of the DeepDicomSort pipeline.

A promising future direction could be to predict the metadata of a scan based on its visual appearance. For example, one could predict the sequence that has been used to acquire a scan (e.g. MPRAGE or MP2RAGE in the case of a T1w scan), or reconstruct the acquisition settings of a scan (e.g. the spin echo time). In this research, we did not consider these types of predictions because we first wanted to focus on the dataset organization, however we think that our method can provide a basis for these types of predictions.

## Conclusion

We developed an algorithm that can recognize T1w, T1wC, T2w, PDw, T2w-FLAIR, DWI, PWI-DSC, and derived brain (MRI) scans with high accuracy, outperforming the currently available methods. We have made our code and trained models publicly available under an Apache 2.0 license.^3^ Using the code and the trained models, one can run the DeepDicomSort pipeline and structure a dataset either according to the BIDS standard or a self-defined layout. We think that scan type recognition is an essential step in any data curation pipeline used in medical imaging. With this method, and by making our code and trained models available, we can automate this step in the pipeline and make working with large, heterogeneous datasets easier, faster, and more accessible.

## Information Sharing Statement

Code and trained models for the algorithms constructed in this paper are publicly available on GitHub under an Apache 2.0 license at https://github.com/Svdvoort/DeepDicomSort. Part of the pre-processing code depends on FSL. Since FSL is only licensed for non-commercial use, (this part of) the code cannot be used in a commercial setting.

All data used in this research is publicly available. The Cancer Imaging Archive collections mentioned are all publicly available at https://www.cancerimagingarchive.net/. The datasets from the Norwegian National Advisory Unit for Ultrasound and Image-Guided Therapy are publicly available at https://www.sintef.no/projectweb/usigt-en/data/. The BITE collection is publicly available at http://nist.mni.mcgill.ca/?page_id=672. The Alzheimer’s Disease Neuroimaging Initiative data is available at http://adni.loni.usc.edu/, after submitting an application which must be approved by the ADNI Data Sharing and Publications Committee.

## Figures and Tables

**Fig. 9:**
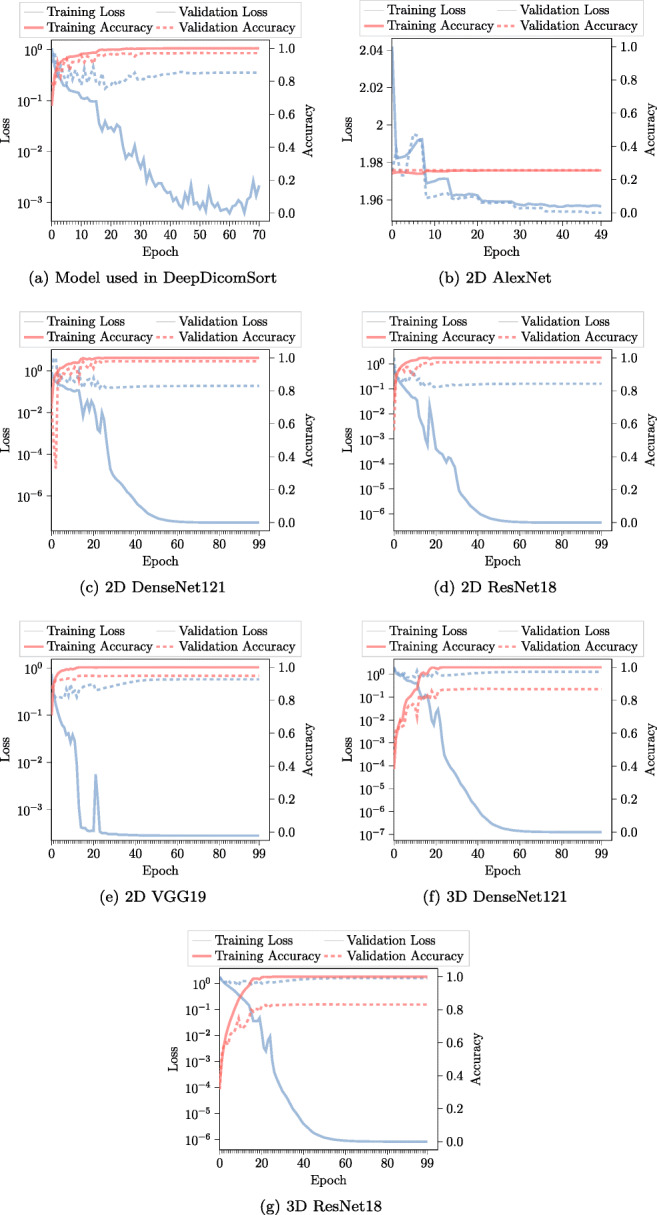
Learning curves of the different network architectures tested in the model parameter selection

**Fig. 10:**
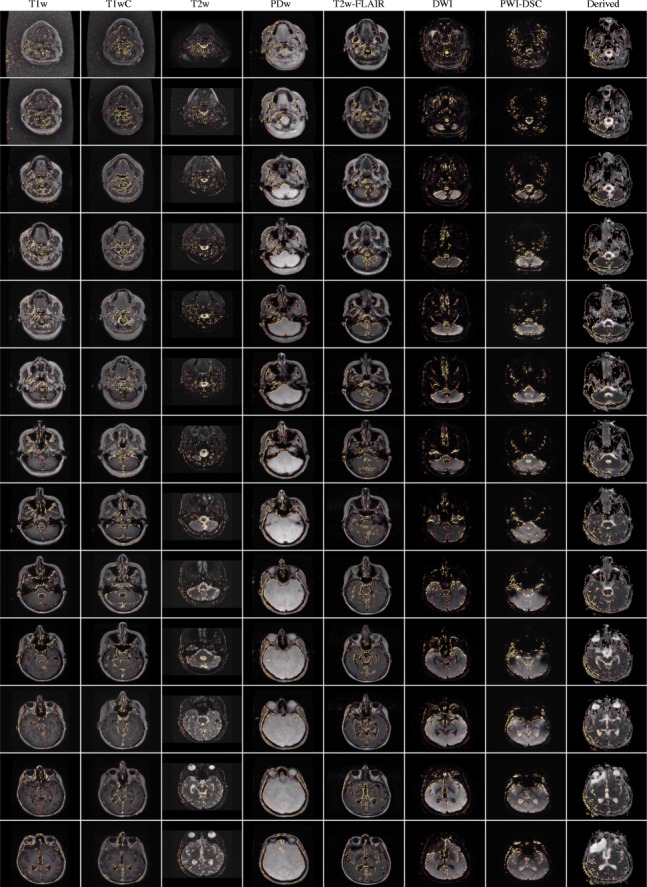
Saliency maps for slices 1 through 13 of the subject from Fig. 1

**Fig. 11:**
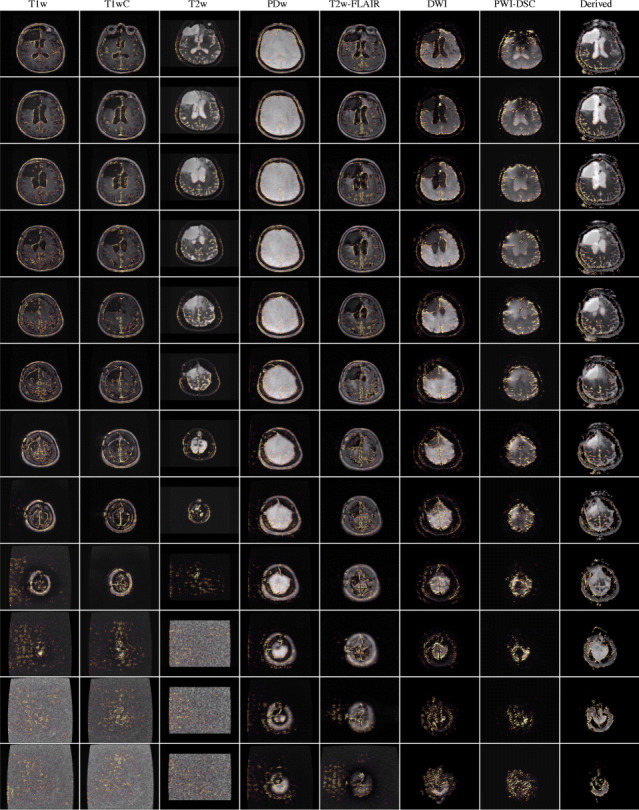
Saliency maps for slices 14 through 25 of the subject from Fig. 1

**Fig. 12:**
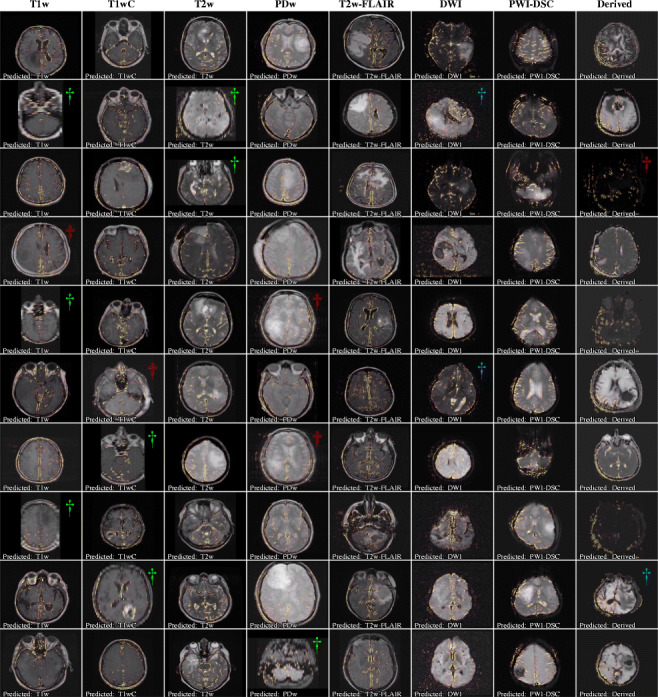
Saliency maps and predicted scan type of randomly drawn samples from the brain tumor test set

**Fig. 13:**
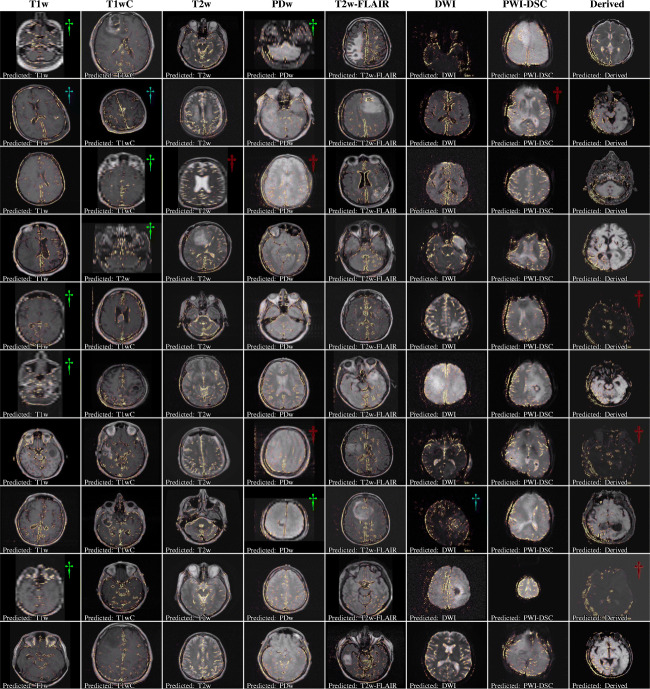
Saliency maps and predicted scan type of randomly drawn samples from the brain tumor test set

**Fig. 14:**
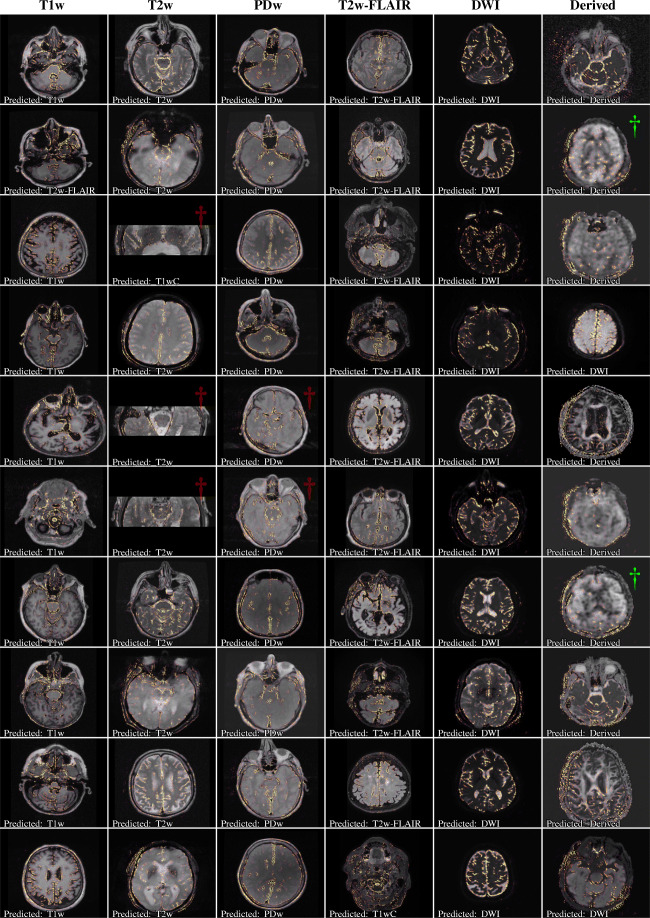
Saliency maps and predicted scan type of randomly drawn samples from the ADNI dataset

**Fig. 15:**
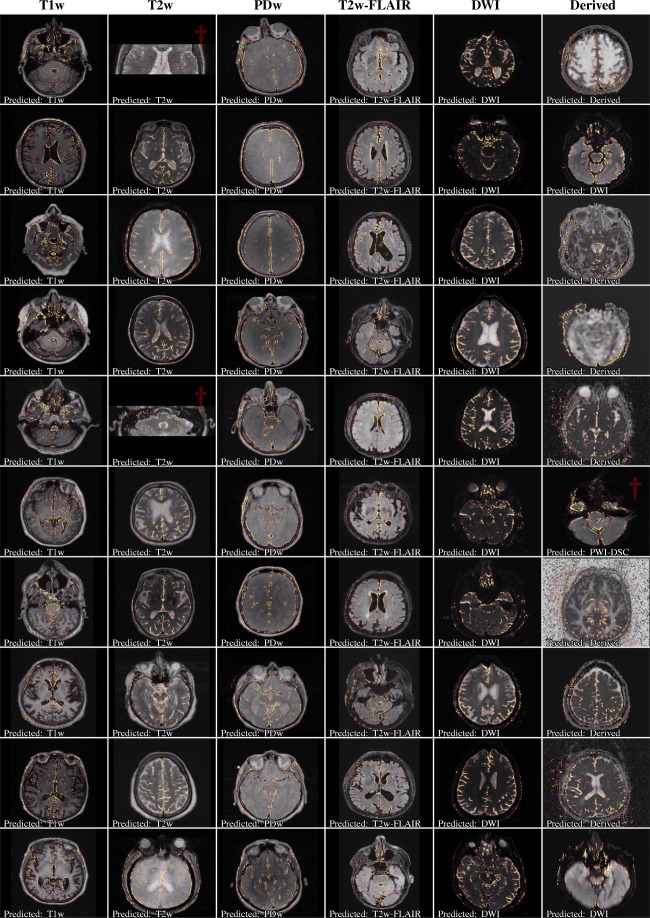
Saliency maps and predicted scan type of randomly drawn samples from the ADNI dataset

**Fig. 16:**
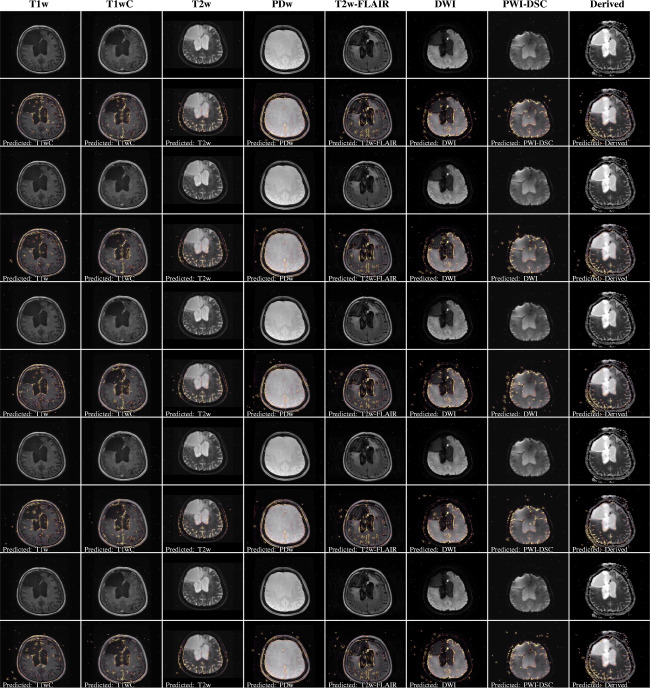
Saliency maps and predicted sacn types of the derived slices from Fig. 1 after randomly setting some pixels to the maximum intensity. Every time the slice with the added noise is shown, followed by the saliency map and predicted scan type for the same slice in the row below

**Fig. 17:**
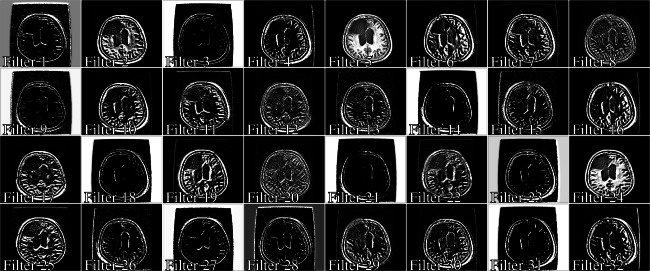
Feature map visualizations of the trained CNN from Experiment I. These visualization were obtained by passing a T1w slice through the network, and showing the results directly after convolutional layer 1. The slice is the same as the one shown in Fig. 1

**Fig. 18:**
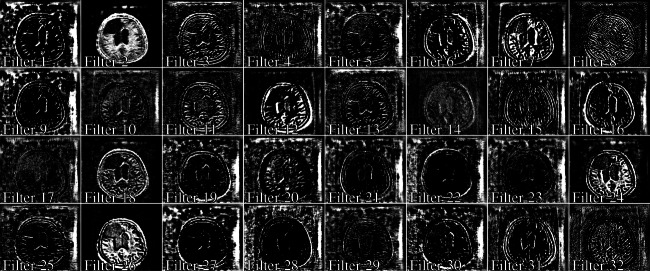
Feature map visualizations of the trained CNN from Experiment I. These visualization were obtained by passing a T1w slice through the network, and showing the results directly after convolutional layer 2. The slice is the same as the one shown in Fig. 1

**Fig. 19:**
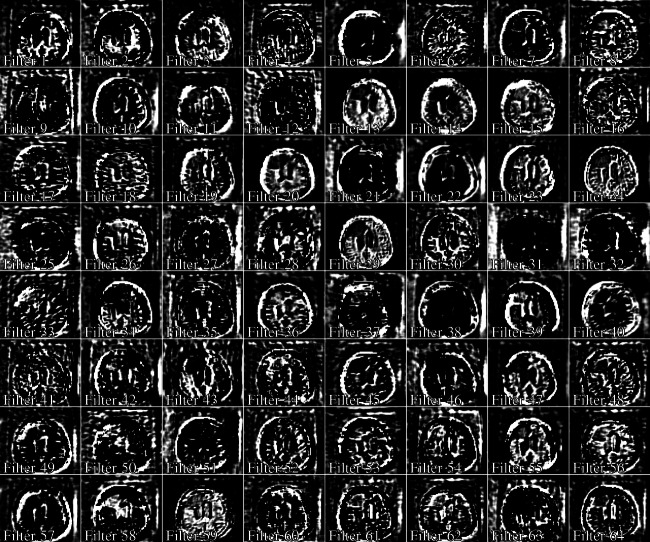
Feature map visualizations of the trained CNN from Experiment I. These visualization were obtained by passing a T1w slice through the network, and showing the results directly after convolutional layer 3. The slice is the same as the one shown in Fig. 1

**Fig. 20:**
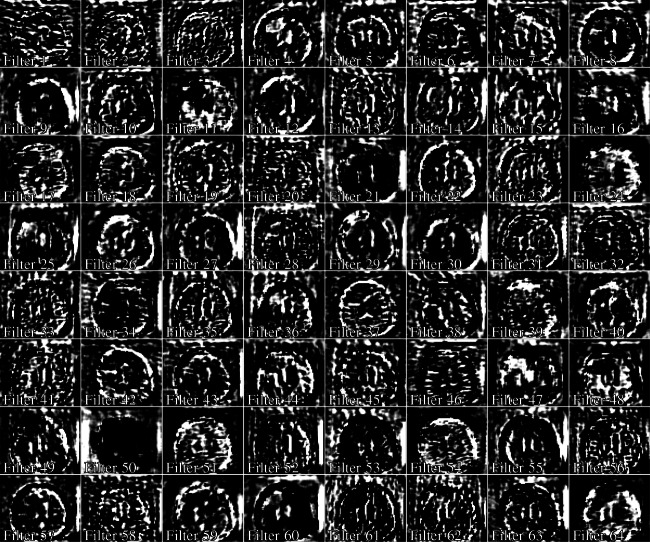
Feature map visualizations of the trained CNN from Experiment I. These visualization were obtained by passing a T1w slice through the network, and showing the results directly after convolutional layer 4. The slice is the same as the one shown in Fig. 1

**Fig. 21:**
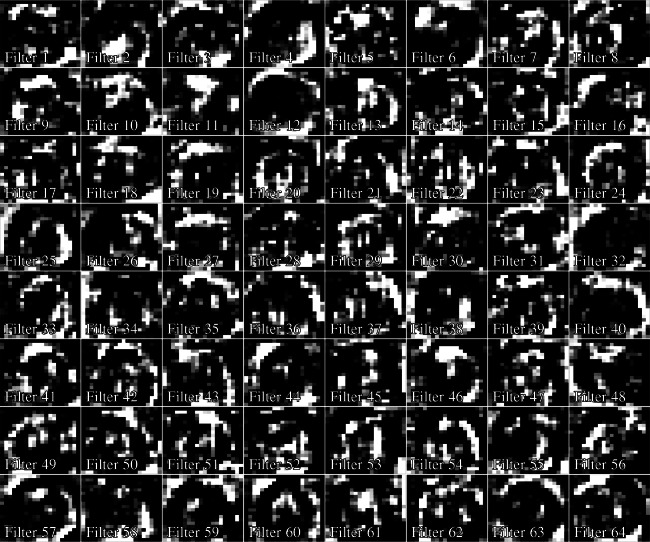
Feature map visualizations of the trained CNN from Experiment I. These visualization were obtained by passing a T1w slice through the network, and showing the results directly after convolutional layer 5. The slice is the same as the one shown in Fig. 1

**Fig. 22:**
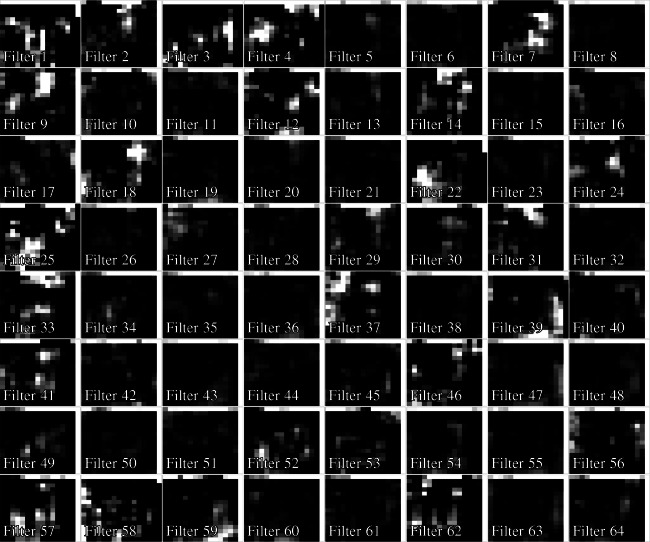
Feature map visualizations of the trained CNN from Experiment I. These visualization were obtained by passing a T1w slice through the network, and showing the results directly after convolutional layer 6. The slice is the same as the one shown in Fig. 1

## Appendix A: Model Parameter Selection

To determine the optimal model parameters (i.e. the CNN architecture, pre-processing settings and optimizer settings) of the CNN used in the DeepDicomSort pipeline, we evaluated the performance of different model parameters on the brain tumor train set, the train set from Experiment I. Before carrying out the experiments, the brain tumor train set was partitioned into a train set and validation set. 85% of the scans was used as a train set and 15% of the scans was used as a validation set. Only one such split was made since training and validating the network for multiple splits would be too time-consuming. During the splitting, all slices of a scan where either all in the train set or all in the validation set to prevent data leakage between the train set and validation set.

We compared five different CNN architectures: the architecture proposed in this paper, Alexnet (Krizhevsky et al. 2012), ResNet18 (He et al. 2016), DenseNet121 (Huang et al. 2017) and VGG19 (Simonyan and Zisserman 2015). For all networks, the same pre-processing approach as described in Section “Pre-Processing” was used, with the optimizer settings as described in Section “Network”. The only difference was that the learning rate reduction was based on the validation loss instead of the training loss. For the VGG19 model, the initial learning rate was lowered to 0.0001, as the model would otherwise get stuck in a poor minimum early in the training stage. Different pre-processing settings (e.g. different normalization settings) and model settings (e.g. learning rate) were tested. However, here we show only the effect of the different architectures using the same pre-processing settings for all models to make a fair comparison and since we obtained the best results using these pre-processing settings.

The learning curves for the different models are shown in Fig. 9. The learning curve for the AlexNet model (Fig. 9b), shows that this model is the only one that was not capable to properly train for the task at hand, probably due to the low number of weights that can be optimized in this model. Except for the AlexNet model, all the other models were able to properly learn, and the final validation accuracy was roughly the same for all models. The DenseNet model achieved the highest validation accuracy of 98%, a full overview of the performance of the different CNN architectures can be found in Table 7. These results show that multiple models work for the problem at hand. Ultimately, we chose to employ our proposed architecture because it is less computationally intensive than the other models. Not only does our model train faster (shown in Table 7), it also requires less time to predict the scan type of new scans, and requires less (GPU) memory. Selecting the least computationally intensive model allows a wider adoption of our tool.

**Table 7:** Overall training accuracy, overall validation accuracy, and the time it took to train each network for the different network architectures test in the model parameter selection

	Train	Validation	Training time (h)
*Our model*	0.999	0.971	14.8
*2D AlexNet*	0.255	0.255	11.0
*2D DensNet121*	1.000	0.980	21.8
*2D ResNet18*	1.000	0.973	26.4
*2D VGG19*	1.000	0.948	34.6
*3D DenseNet121*	1.000	0.868	22.9
*3D ResNet18*	1.000	0.832	27.7

A train/validation split of the brain tumor train set was used to determine the performance

We also trained two 3D models to compare their performance with the 2D models. In the case of the 3D models, most of the pre-processing steps were kept the same, apart from the slice extraction. Instead of extracting 25 slices, 3D patches with a size of 90 × 90 × 15 voxels were extracted. A maximum of 10 patches per scan were extracted, in such a way that they covered as much of the (geometrical) center of the scan as possible to ensure that the patches contained brain and not just background. We trained a 3D ResNet18 and a 3D DenseNet121; the learning curves can be seen in Fig. 9f and g. These 3D architectures achieved a lower validation accuracy than their 2D counterparts, 0.87 versus 0.98 for the DenseNet model and 0.83 versus 0.97 for the ResNet model. These results justified our choice for a 2D model, which not only achieved a higher accuracy but was also less computationally intensive.

## Appendix B: Confusion Matrices

The confusion matrices for Experiment I (Table 8) and Experiment II (Table 9), which show the relation between the ground truth scan type and the predicted scan type.

**Table 8:** Confusion matrix of results from Experiment I

Ground truth	Predicted								
		*T1w*	*T1wC*	*T2w*	*PDw*	*T2w-FLAIR*	*DWI*	*PWI-DSC*	*Derived*
	*T1w*	433	2	0	1	0	0	0	0
	*T1wC*	2	608	0	0	0	0	0	0
	*T2w*	1	2	292	0	0	0	0	0
	*PDw*	0	0	0	181	0	0	0	0
	*T2w-FLAIR*	18	0	0	0	238	0	0	0
	*DWI*	0	0	0	0	0	344	2	1
	*PWI-DSC*	0	0	0	0	0	0	87	0
	*Derived*	0	0	1	0	0	0	0	156

**Table 9:** Confusion matrix of results from Experiment II

Ground truth	Predicted								
		*T1w*	*T1wC*	*T2w*	*PDw*	*T2w-FLAIR*	*DWI*	*PWI-DSC*	*Derived*
	*T1w*	2655	1	0	0	0	0	0	0
	*T1wC*	0	0	0	0	0	0	0	0
	*T2w*	0	34	2140	44	0	0	0	0
	*PDw*	0	0	2	1067	0	0	0	0
	*T2w-FLAIR*	6	5	0	1	468	12	0	0
	*DWI*	0	0	0	0	0	557	3	0
	*PWI-DSC*	0	0	0	0	0	0	0	0
	*Derived*	0	0	1	0	0	3	0	228

## Appendix C: Predictive Performance on Per-Slice Basis

Table 10 shows the accuracy of the CNNs from Experiment I and Experiment II on a per-slice basis instead of on a per-scan basis. These results are obtained by comparing the predicted class of a slice directly with the ground truth class of that slice before the individual slice predictions are combined by a majority vote to obtain the scan type.

**Table 10:** Overall accuracy and per-class accuracy achieved by DeepDicomSort in Experiment I and Experiment II on a per-slice basis

	Experiment I	Experiment II
*Overall*	0.934	0.851
*T1w*	0.942	0.814
*T1wC*	0.940	N/A
*T2w*	0.926	0.894
*PDw*	0.905	0.914
*T2w-FLAIR*	0.879	0.592
*DWI*	0.985	0.943
*PWI-DSC*	0.925	N/A
*Derived*	0.990	0.908

## Appendix D: Time Comparison Between DeepDicomSort, HeuDiConv and Manual Sorting

We estimated the potential time that can be saved by using DeepDicomSort to sort a dataset instead of doing so by hand or using HeuDiConv. We did so by assuming the hypothetical situation where one has an automated tool that requires the T1wC and T2w-FLAIR scans as inputs, and we compared the time needed to find the T1wC and T2w-FLAIR scans for all subjects and sessions in the brain tumor test set. The manual sorting was simulated by iterating over all scans in a session in random order until either the T1wC and T2w-FLAIR scans were found or until there were no more scans to check. The sorting of the dataset using HeuDiConv or DeepDicomSort was simulated by first iterating over all scans that were predicted as a T1wC or T2w-FLAIR by these methods, and checking whether that prediction was correct. If the predicted scan type was incorrect, the same approach as for the manual sorting was followed to find the correct scans. We assumed that, on average, a human required 25 seconds per scan to visually identify the correct scan type. By multiplying this time per scan with the total number of scans that were iterated over, we obtained an estimate for the total time taken by each method to find the T1wC and T2w-FLAIR scans. We used the brain tumor test set to evaluate the timing results, since HeuDiConv was only optimized for the brain tumor dataset.

### D.1 Results

The time required to identify the T1wC and T2w-FLAIR scan for each session in the brain tumor test set by hand was estimated to be 29.0 hours. The estimated time required to check and correct the automated scan type recognition by HeuDiConv was 35.7 hours, which excludes the time required to construct the heuristic. If the automated scan type recognition was done by DeepDicomSort instead, we estimated that 12.3 hours of manual time were required. The time required to run the DeepDicomSort pipeline on the dataset was 61.5 minutes using an Intel Xeon Processor E5-2690 v3 for pre-processing and post-processing, and an Nvidia Tesla K40m GPU to classify the samples using the CNN. If the scans identified by DeepDicomSort were used without a manual check, in which case the total sorting time was only 61.5 minutes, 527 scans would have been correctly identified. Four scans were incorrectly identified as a T1wC or T2w-FLAIR scan, for one session the T1wC would not have been found, and for 8 sessions the T2w-FLAIR would not have been found.

It should be noted that with the automated methods (DeepDicomSort and HeuDiConv), one gets a fully sorted dataset, whereas the sorting by hand still requires the sorting of the scans that were not yet identified.

## Appendix E: Saliency Map for Full Scan

Figures 10 and 11 show the saliency maps for all 25 slices from the scans of the example subject from Fig. 1. The CNN seems to focus on the same features as in Fig. 7, mostly on the ventricles and on the CSF at the edges of the brain. In the superior slices of the scan, it can be seen that the presence of a tumor does not disrupt the CNN. Although it looks at the edge of the tumor, it does not put a lot of focus on the tumor itself. For the most superior slices of T1w, T1wC and T2w it can be seen that when the brain is no longer present in the slice the CNN loses it focus and seems to look randomly throughout the slice.

## Appendix F: Saliency Maps for Additional Examples

### F.1 Random Samples from the Brain Tumor Test Set

To show the robustness of our method to differences in scan appearance, as well as to imaging artifacts, we have randomly selected 20 slices of each scan type from the brain tumor test set. All of these slices were then passed through the CNN, and we determined the saliency maps along with the predicted class of each slice. This is the prediction based on the slice itself, and thus before the majority vote. The saliency maps and predicted sacn types are shown in Figs. 12 and 13. We have highlighted slices that contain imaging artifacts (), have a poor image quality (), and subjects with a large head tilt (). These saliency maps show that the CNN is quite robust to the presence of a tumor, the presence of imaging artifacts, or poor image quality, in most cases the CNN still predicts the correct scan type.

### F.2 Random Samples from ADNI Dataset

The same approach as in Appendix F.1 has been applied to show the saliency maps from random samples of the ADNI dataset. In this case, the saliency maps were derived using the trained model from Experiment II instead of Experiment I. Once again the saliency maps and the predicted scan type are shown in Figs. 14 and 15. We have highlighted slices that contain imaging artifacts, including hippocampus scans with a limited field of view, (), have a poor image quality (), and subjects with a large head tilt ().

### F.3 Robustness Against Bright Noise

To test the effect of potential bright spots in the scan, we performed an experiment where random bright spots were introduced in the slices from Fig. 1. Within each slice 0.5% of voxels were randomly chosen, and the intensity of these voxels was set to the maximum intensity of the slice. We then determined the saliency maps for these slices and the predicted scan type, the results are shown in Fig. 16.

These results show that our method is quite robust against bright spots in a scan. Only for the T1w and PWI-DSC scans there were slices that were misclassified. In the case of the T1w slice, there were two out of five slices that were predicted to be T1wC. This is most likely caused by the CNN having learned that a T1w and T1wC scan have a similar appearance in general, but that the T1wC scan has brighter spots. In two cases the PWI-DSC slice was misclassified as a DWI. Probably this is caused by the CNN seeing the random brightness spots outside the skull as imaging artifacts, which often show up in DWI scans and less so in PWI-DSC scans. Although the CNN misclassified the T1w and PWI-DSC slices in some cases, when bright spots were introduced on all 25 slices of the T1w and PWI-DSC scans (randomly for each slice) and then passed through the network, the CNN still predicted the correct scan type of the scan after the majority vote.

## Appendix G: Feature Map Visualizations

Figures 17 through 22 show the feature maps of all filters of each convolutional layer for the T1w slice shown in Fig. 1. It can be seen that some filters mainly identvify the skull (for example, filter 1 from convolutional layer 1), whereas other filters seem to focus on specific structure (for example, filter 4 from convolutional layer 1, which seems to identify gray matter).

## Footnotes

1 https://github.com/Svdvoort/DeepDicomSort

2 https://github.com/nipy/heudiconv

3 https://github.com/Svdvoort/DeepDicomSort
